# Prognostic implications of carboxyl-terminus of Hsc70 interacting protein and lysyl-oxidase expression in human breast cancer

**DOI:** 10.4103/1477-3163.72505

**Published:** 2010-11-12

**Authors:** Neill Patani, Wen Jiang, Robert Newbold, Kefah Mokbel

**Affiliations:** 1 Department of Breast Surgery, The London Breast Institute, The Princess Grace Hospital, London; 2 Metastasis & Angiogenesis Research Group, University Department of Surgery, Cardiff University, Cardiff, Wales, London; 3 The Brunel Institute of Cancer Genetics and Pharmacogenomics, London; 4 Department of Breast Surgery, St. George’s University of London

**Keywords:** Breast cancer, prognosis, recurrence, survival, tumor suppressor

## Abstract

**Background:**

Ubiquitin modification of proteins influences cellular processes relevant to carcinogenesis. CHIP (carboxyl-terminus of Hsc70-interacting protein) is a chaperone-dependent E3 ubiquitin ligase, regulating the stability of heat shock protein 90 (HSP90) interacting proteins. CHIP is implicated in the modulation of estrogen receptor (ESR1) and Her-2/neu (ERBB2) stability. LOX (lysyl-oxidase) serves intracellular roles and catalyses the cross-linking of extracellular matrix (ECM) collagens and elastin. LOX expression is altered in human malignancies and their peri-tumoral stroma. However, paradoxical roles are reported. In this study, the level of mRNA expression of CHIP and LOX were assessed in normal and malignant breast tissue and correlated with clinico-pathological parameters.

**Materials and Methods:**

Breast cancer (BC) tissues (*n* = 127) and normal tissues (*n* = 33) underwent RNA extraction and reverse transcription; transcript levels were determined using real-time quantitative PCR and normalized against CK-19. Transcript levels were analyzed against TNM stage, nodal involvement, tumor grade and clinical outcome over a ten-year follow-up period.

**Results:**

CHIP expression decreased with increasing Nottingham Prognostic Index (NPI): NPI-1 vs. NPI-3 (12.2 vs. 0.2, *P* = 0.0264), NPI-2 vs. NPI-3 (3 vs. 0.2, *P* = 0.0275). CHIP expression decreased with increasing TNM stage: TNM-1 vs. TNM-2 (12 vs. 0, *P* = 0.0639), TNM-1 vs. TNM-2-4 (12 vs. 0, *P* = 0.0434). Lower transcript levels were associated with increasing tumor grade: grade 1 vs. grade 3 (17.7 vs. 0.3, *P* = 0.0266), grade 2 vs. grade 3 (5 vs. 0.3, *P* = 0.0454). The overall survival (OS) for tumors classified as ‘low-level expression’, was poorer than those with ‘high-level expression’ (118.1 vs. 152.3 months, *P* = 0.039). LOX expression decreased with increasing NPI: NPI-1 vs. NPI-2 (3 vs. 0, *P* = 0.0301) and TNM stage: TNM-1 = 3854639, TNM-2 = 908900, TNM-3 = 329, TNM-4 = 1.232 (*P* = NS).

**Conclusion:**

CHIP expression is associated with favorable prognostic parameters, including tumor grade, TNM stage and NPI. CHIP expression predicts OS. LOX expression is associated with improved NPI. In addition to their prognostic utility, mechanistic insights into tumor suppressor function may offer potential therapeutic strategies.

## INTRODUCTION

Ubiquitin modification of proteins influences diverse cellular processes relevant to cancer pathogenesis. These include: targeting of proteins for degradation, endocytosis, kinase activation, sub-nuclear trafficking, ribosome modification and DNA repair.[1,2] Protein stability is regulated by ubiquitination and subsequent degradation via the proteasome or lysosome.[3] Ballinger *et al*. described a highly conserved chaperone interacting protein CHIP (carboxyl-terminus of Hsc70 interacting protein), expressed primarily in striated muscle and brain. CHIP is a chaperone-dependent E3 ubiquitin ligase, implicated in the ubiquitination and proteasomal degradation of several HSP90 interacting proteins.[4,5] CHIP possesses a carboxyl-terminal U-box domain, which interacts with ubiquitin-conjugating enzymes and mediates ubiquitin ligase activity, and an amino-terminal tetratricopeptide repeat (TPR) motif, which interacts with molecular chaperones such as HSP/Hsc70 and HSP90 and antagonizes their substrate chaperone functions.[5,6] This promotes ubiquitination and degradation of substrate proteins, such as the glucocorticoid receptor, the cystic fibrosis transmembrane conductance regulator and c-Raf (RAF1) kinase.[5,7,8] CHIP is implicated in post-translational quality-control activity, triaging and partitioning proteins towards either folding or degradation pathways.[6,9,10]

In a recent study, CHIP has been implicated in regulating the stability and turnover of estrogen receptor alpha (ESR1). Following CHIP transfection, BC cell lines demonstrated increased ESR1 proteasomal degradation and decreased ESR1-mediated gene transcription compared to those with CHIP depletion.[11] ESR1 is maintained in a ligand-binding conformation by HSP90-based chaperones.[12] and HSP90 inhibitors, such as geldanamycin (GA), enhance the ESR1-CHIP interaction and promote degradation through the ubiquitin-proteasome pathway.[13,14] In this way, CHIP can influence ESR1-receptor profiles and hormone responsiveness. Similarly, Yi *et al*. have demonstrated that the histone deacetylase inhibitor suberoylanilide hydroxamic acid (SAHA) acetylates and inactivates HSP90, inducing ESR1 degradation via the CHIP-mediated ubiquitin-proteasome pathway, effectively inhibiting proliferation and inducing apoptosis in MCF-7 cells.[15] SAHA has been the subject of early phase clinical trials for hematological and solid human cancers.[16,17]

The stability of mature Her-2/neu(ERBB2) requires association with the HSP90 chaperone. In-vitro co-transfection studies of CHIP and ERBB2 have implicated CHIP in the ubiquitination and down-regulation of ERBB2.[18] CHIP appears to induce a HSP/Hsc70 pro-degradation chaperone complex to associate with ERBB2. CHIP has also been found to mediate ERBB2 degradation induced by the flavonoid Quercetin (3, 5, 7, 30, 40-pentahydroxyflavone) in a study of BC cell lines.[19] Quercetin appears to enhance the binding activity of CHIP, encouraging ERBB2 ubiquitination and proteasomal degradation. In addition, GA promotes ERBB2-HSP90 chaperone complex dissociation followed by association with HSP70-CHIP, which facilitates degradation via the ubiquitin-proteasome pathway.[18,20–24] The chaperone activity of HSP90 requires ATP, depletion of which disrupts the stabilization of ERBB2 by HSP90 leading to dissociation and degradation.[25] Indeed, GA has been demonstrated to specifically bind to HSP90 and inhibit its ATPase activity.[26] Targeting ERBB2 for degradation in this manner differs from the mechanism of action of the humanized monoclonal antibody Trastuzumab (Herceptin). A combination of Herceptin and the HSP90 inhibitor 17-allylamino-geldanamycin (17-AAG) enhances ubiquitination and lysosomal degradation of ERBB2 and cytotoxicity in ERBB2-overexpressing BC cells. Trastuzumab and 17-AAG mediate recruitment of different E3 ubiquitin ligases, casitas B-lineage lymphoma (Cbl) and CHIP respectively, to ERBB2 with a synergistic effect on reducing proliferation and promoting apoptosis.[27,28] Clinical trials are currently under-way evaluating 17-AAG in BC.

Lysyl-oxidase (LOX) has both intracellular and extracellular functions relevant to carcinogenesis. LOX activation depends on the transport of internalized copper to the trans-golgi network.[29] LOX is assembled as a pre-proenzyme, which is glycosylated before secretion. The pro-enzyme (Pro-LOX) undergoes proteolytic cleavage to the functional enzyme (LOX) and the lysyl oxidase pro-peptide (LOX-PP).[30] LOX functions primarily as an ECM-modulating enzyme catalyses the cross-linking of collagens and elastin, thereby influencing insoluble matrix deposition and tensile strength structural integrity. LOX is essential for normal connective tissue, embryonic development and wound healing.[30,31] Interactions between cancer cells and the ECM within their immediate micro-environment are likely to influence cellular proliferation, local invasion and metastatic potential. In addition, LOX has been implicated in diverse biological activities including cell motility and migration, chemotaxis, cell adhesion, differentiation and transcriptional gene regulation.[32–36]

The tumor suppressor function of LOX was initially demonstrated in ras-transformed fibroblasts, lacking LOX transcription and activity. LOX was identified as a ras-recision gene, able to reverse the oncogenic activities of ras.[37] Other studies have also demonstrated in-vitro tumor suppressor function.[38–41] LOX down-regulation has been identified in several human malignancies, including: colorectal cancer, bronchogenic carcinoma, gastric cancers, head and neck squamous cell carcinomas, and primary and metastatic prostate tumors.[42–46] Bissell *et al*. characterized malignant breast tumors by the loss of normal tissue architecture and cell-ECM interactions.[47] In keeping with this, LOX expression has been reported to be maximal in the fibrotic stroma surrounding in-situ ductal carcinoma (DCIS) and in the reactive fibrosis, facing the invasion front of infiltrating tumors. Formation of a scar-like peri-tumor barrier may represent a host response to limit invasion. Comparatively lower expression of LOX was associated with invading tumors, resulting in a loose scirrhous stroma and the deposition of non-cross-linked matrix proteins susceptible to degradation.[48] Decitre *et al*. have also reported the localization of a LOX-like protein within the early stromal reaction of DCIS.[49]

In this study, the expression profile of CHIP and LOX is assessed in a cohort of women with BC. Transcript levels are evaluated against established pathological parameters and clinical outcome over a ten-year follow-up period.

## MATERIALS AND METHODS

Sample collection and patient recruitment occurred between 1991 and 1997. Institutional guidelines, including ethical approval and informed consent, were followed. BC tissues (*n* = 127) and normal background tissues (*n* = 33) were collected immediately after excision during surgery and stored at −80° C until use. A consultant pathologist examined hematoxylin- and eosin-stained frozen sections to verify the presence of tumor cells in the collected samples. Normal tissue was derived from the background breast parenchyma of BC patients within the study group. Medical notes and histology reports were used to extract clinico-pathological information [Table 1]. A customized database was established to record the data.

**Table 1:** Clinical and pathological data

Parameter	Category	Number
Node status	Node positive	54
	Node negative	73
Tumor grade	1	24
	2	43
	3	58
Tumor type	Ductal	98
	Lobular	14
	Medullary	2
	Tubular	2
	Mucinous	4
	Others	7
TNM staging	1	70
	2	40
	3	7
	4	4
Outcome	Disease-free	90
	Alive with metastasis	7
	With local recurrence	5
	Died of breast cancer	16
	Died of unrelated disease	9

Note: missing values reflect discarded/uninterpretable values

RNA extraction kits and reverse transcription kits were obtained from Sigma-Aldrich Ltd (Poole, Dorset, England, UK). The PCR primers were designed using Beacon Designer (Palo Alto, CA, USA) and synthesized by Sigma-Aldrich. Custom made hot-start master-mix for quantitative PCR was obtained from Abgene (Surrey, England, UK). Frozen sections of tissue were cut at a thickness of 5–10 mm and kept for routine histological analysis. Additional 15–20 sections were mixed and homogenized using a hand-held homogenizer in ice-cold RNA extraction solution. The concentration of RNA was determined using ultraviolet spectrophotometry. Reverse transcription was carried-out using a reverse transcription kit with an anchored olig (dT) primer supplied by Abgene, using 1 mg of total RNA in a 96-well plate. The quality of cDNA was verified using ß-actin primers [Table 2].

**Table 2:** Primer sequences

Primers for β-Actin	
ATGATATCGCCGCGCTCGTC	
CGCTCGGTGAGGATCTTCA	
Primers for CK-19	
CAGGTCCGAGGTTACTGAC	
ACTGAACCTGACCGTACACACTTTCTGCCAGTGTGTCTTC	
Primers for CHIP	
AGTGGCATCACCTACGAC	CHIPF1
ACTGAACCTGACCGTACAAAGTTGGGGATGAGCTGT	CHIPZR1
GATGGAGAGCTATGATGAGG	CHIPF2
ACTGAACCTGACCGTACAGCTTCTTCTTCGCGATTC	CHIPZR2
Primers for LOX	
CCTGTGACTATGGCTACCAC	LYLOXF1
ACTGAACCTGACCGTACATGTCTGCACCATAGGTATCA	LYLOXZR1
TACTTATGAAAGGCCCAGAC	LYLOXF2
ACTGAACCTGACCGTACATACATGGACATCTTCTGCAC	LYLOXZR2

The level of CHIP and LOX transcripts from the above-prepared DNA were determined using real-time quantitative PCR based on the amplifluor technology, modified from a method reported previously.[50,51] The PCR primers were designed using Beacon Designer software, but to the reverse primer an additional sequence, known as the Z sequence (5′-ACTGAACCTGACCGTACA-3′), which is complementary to the universal Z-probe (Intergen Inc., Oxford, UK) was added. The product expands one intron. The primers used for each are detailed in Table 2. The reaction was carried-out using Hot-start Q-master mix (Abgene), 10 pmol of specific forward primer, 1 pmol reverse primer that had Z sequence, 10 pmol of FAM (fluorogenic reporter dye, carboxy fluorescein) tagged probe (Intergen Inc.) and cDNA from 50 ng of RNA. The reaction was carried-out using the IcyclerIQ (Bio-Rad Ltd, Hemel Hempstead, England, UK), which is equipped with an optic unit that allows real-time detection of 96 reactions, under the following conditions: 94° C for 12 min and 50 cycles of 94° C for 15 s, 55° C for 40 s and 72° C for 20 s. The levels of the transcript were generated from a standard that was simultaneously amplified with the samples. The levels of gene-expression were then normalized against the housekeeping gene CK-19, which was already quantified in these specimens, to correct for varying amounts of epithelial tissue between samples.[52] The primers used for CK-19 are detailed in Table 2. Each PCR run included a negative control without a template and a known cDNA reference sample as a positive control.

The Mann-Whitney *U*-test (comparison of median copy number) and two-sample *t*-test (comparison of mean copy number) were used for statistical analysis of absolute and normalized gene copy number. The transcript levels within the BC specimens were compared to normal background tissues and analyzed against conventional pathological parameters and clinical outcome over a ten-year follow-up period. In each case, the true copy number was used for statistical analysis and hence the samples were not classified as positive or negative. The statistical analysis was carried-out using Minitab version 14.1 (Minitab Ltd. Coventry, England, U.K.) using a custom written macro (Stat 2005.mtw). For purposes of the Kaplan-Meier survival analysis, the samples were divided arbitrarily into two groups, ‘high transcript level’ or ‘low transcript level’, for each gene. The cut-off was guided by the NPI value with which the value of the moderate prognostic group was used as the dividing-line at the start of the test. Survival analysis was performed using SPSS version 12.0.1 (SPSS Inc. Chicago, IL, USA). NPI = tumor size (cm) × 0.2 + lymph node stage (1 or none of the nodes affected; 2 to 3 nodes affected; more than 3 nodes affected) + Grade (1–3, Scarff-Bloom-Richardson). NPI scores were classified into three groups: < 3.4 = NPI-1, 3.4–5.4 = NPI-2, > 5.4 = NPI-3. Within tumor samples, ESR1 and ERBB2 status were classified according to the transcript copy number per 50 ng (nanograms) of RNA: < 1 = negative, ≥ 1 = positive.

## RESULTS

CHIP expression profiles were determined in absolute terms and normalized against CK-19, in order to correct for varying amounts of epithelial tissue between samples [Table 3]. CHIP was found to be expressed in both normal/benign breast tissue and BC specimens. Analysis of 25 paired samples demonstrated a trend for levels to be higher in normal/benign tissue compared to tumor samples, although this did not reach statistical significance [Table 4]. The expression of CHIP mRNA was demonstrated to decrease with increasing NPI: NPI-1 vs. NPI-3 (normalized median copy number 12.2 vs. 0.2, *P* = 0.0264), NPI-2 vs. NPI-3 (normalized median copy number 3 vs. 0.2, *P* = 0.0275). The expression of CHIP mRNA was also demonstrated to decrease with increasing TNM stage: TNM-1 vs. TNM-2 (normalized median copy number 12 vs. 0, *P* = 0.0639), TNM-1 vs. TNM-2–4 (normalized median copy number 12 vs. 0, *P* = 0.0434). In addition, lower transcript levels were also significantly associated with increasing tumor grade: grade 1 vs. grade 3 (normalized median copy number 17.7 vs. 0.3, *P* = 0.0266) and grade 2 vs. grade 3 (normalized median copy number 5 vs. 0.3, *P* = 0.0454). With regard to receptor profiles, there was a trend for ESR1 expression to fall with increasing CHIP transcript levels: ESR1-negative vs. ESR1-positive (normalized mean copy number 104280 vs. 156, *P* = 0.17). A similar trend was noted for ERBB2: ERBB2-negative vs. ERBB2-positive (normalized mean copy number 86750 vs. 10.4, *P* = 0.17; normalized median copy number 3 vs. 0, *P* = 0.0620).

**Table 3:** Summary of expression profiles for the overall cohort, followed by sub-group analysis for tumor specimens and benign specimens. Values represent the true-copy number of mRNA transcripts normalized against CK-19, expressed as mean (median)

	Overall	Tumor	Benign
CHIP	48920 (2)	62445 (3)	4541 (2)
LOX	2036475 (1)	2333742 (1)	1282706 (0)

**Table 4:** Summary of expression profiles for paired tumor and benign specimens (n = 25). Values represent the truecopy number of mRNA transcripts normalized against CK-19, expressed as mean (median); Two-sample t-test for comparison of means and Mann–Whitney U-test for comparison of medians

	Tumor	Benign	Two-sample t-test	Mann–Whitney U-test
CHIP	1612 (0.1)	4682 (1.3)	*P* = 0.40 (NS)	*P* = 0.11 (NS)
LOX	2128 (0)	1334014 (0)	*P* = 0.16 (NS)	*P* = 0.38 (NS)

**Figure 1:**
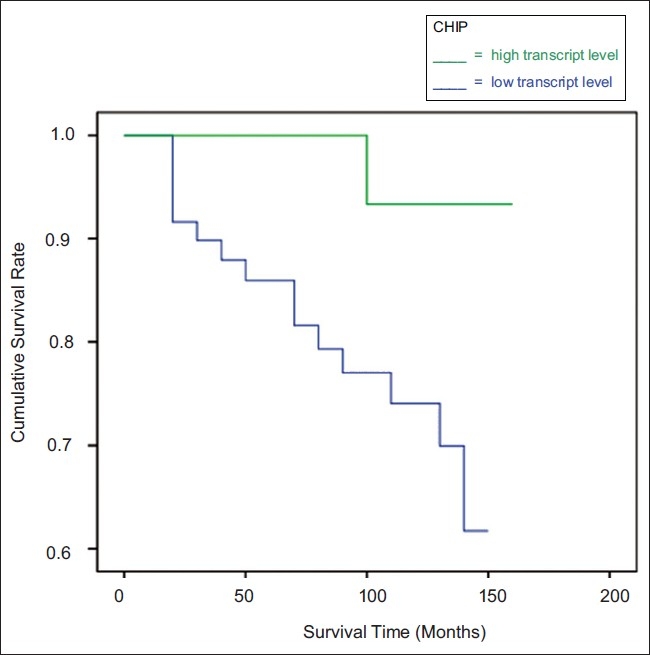
Kaplan Meier Overall Survival curves for CHIP, normalized against CK-19. Survival times are expressed as mean (95% Confidence Interval) months. High level: 152.3 (145.1-159.4), Low level: 118.1 (106.2-130.0), p=0.039

After a median follow-up of ten years, the OS curves for women with tumors, which were classified as having ‘low-levels’ of CHIP transcript, was found to differ significantly from that of their ‘high-level’ counterparts, Figure 1. The survival curves demonstrate that lower levels of CHIP were of utility in predicting poorer OS (*P* = 0.039).

The LOX expression profiles were also determined in absolute terms and normalized against CK-19 [Table 3]. LOX was found to be expressed in both normal/benign breast tissue and BC specimens. Analysis of 25 paired samples demonstrated a trend for levels to be higher in normal/benign tissue compared to tumor samples, although this did not reach statistical significance [Table 4]. The expression of LOX mRNA was demonstrated to decrease with increasing NPI: NPI-1 vs. NPI-2 (normalized median copy number 3 vs. 0, *P* = 0.0301). LOX expression was found to be increased in early stage tumors (TNM-1 mean normalized copy number = 3854639) and decreased in more advanced tumors (TNM-2 = 908900, TNM-3 = 329, TNM-4 = 1.232); however, this trend did not reach statistical significance.

**Figure 2:**
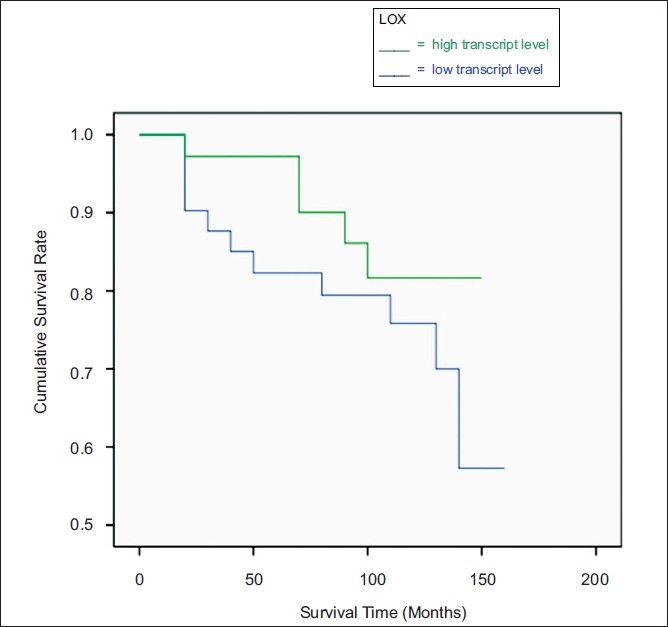
Kaplan Meier Overall Survival curves for LOX, normalized against CK-19. Survival times are expressed as mean (95% Confidence Interval) months. High level: 130.3 (118.9-141.6), Low level: 124.6 (108.3-140.9), *P*=0.225

The OS curve for women with tumors, which were classified as having ‘high-levels’ of LOX transcript was not found to differ significantly from that of their ‘low-level’ counterparts, Figure 2.

## DISCUSSION

CHIP expression was found to be significantly associated with more favorable prognostic parameters, including tumor grade, TNM stage and NPI. Furthermore, CHIP expression emerged as a significant predictor of OS. Our results are consistent with the notion that CHIP functions as a tumor suppressor. However, the underlying mechanisms remain to be elucidated. CHIP has been implicated in regulating the stability and turnover of ESR1 in BC cell lines, effectively inhibiting proliferation and inducing apoptosis.[11,15] Hence, CHIP may be exerting its suppressor activity by regulating ESR1 receptor profiles and hormone responsiveness. Similarly, CHIP expression has been associated with the ubiquitination and down-regulation of ERBB2.[18] The manipulation of innate cellular processes, which determine protein stability and turnover, such as the ubiquitin-proteosome pathway, may provide novel therapeutic strategies. Recent studies have identified a role for CHIP in regulating the association of HSP70/HSP90 chaperones with ESR1 and ERBB2 and receptor down-regulation induced by inhibitors of HSP90, such as GA and SAHA. Following CHIP association, receptor down-regulation is mediated by ubiquitination and subsequent lysosomal and proteasomal degradation. In keeping with this, our study identified a trend for ESR1 and ERBB2 expression to fall with increasing CHIP transcript levels, although this failed to reach statistical significance. Hence, the biological or pharmacological enhancement of specific CHIP interactions may provide rational therapeutic strateg for down-regulating ESR1 and/or ERBB2 activity and inhibiting the proliferation of BC cells. CHIP may not be unique in down-regulating such receptors and further study of related ubiquitin ligases is warranted.[25] In addition to strategies to enhance CHIP expression and activity, development of potent HSP90 inhibitors is required in order to promote dissociation of the ERBB2-chaperone complex. These may provide tailored adjuncts to established ERBB2-targeted therapeutics such as Herceptin, which induce an ERBB2-Cbl interaction resulting in ubiquitination and degradation.[28,53] Combined recruitment of distinct ubiquitin ligases, such as CHIP and Cbl, may be particularly effective in resistant or recurrent cases and could improve toxicity profiles by permitting dose reduction of Herceptin.

LOX expression was demonstrated to decrease with increasing NPI and TNM stage. Our data support the tumor suppressor function of LOX in solid human malignancies.[42–46] However, the underlying mechanisms have been subject to much debate. The peritumoral stromal reaction is likely to have a significant impact on tumor progression. A ‘permissive’ ECM may facilitate tumor development and ‘loosening’ may promote local invasion, angiogenesis and metastases. On the other hand, an ‘inhibitory’ ECM may impede tumor development and progression by a ‘barrier function’, restricting local invasion, angiogenesis and metastases.[48] The complexity and biological relevance of the stromal reaction remains poorly understood. However, a number of studies have identified differential expression of LOX in human cancers and their stromal reaction. Within the extracellular space, LOX effectively contributes to the structural integrity by cross-linking collagens and elastin, and is likely to play a dynamic role in ECM remodelling. Hence, tumor suppressor function may be mediated by the formation of an ‘inhibitory’ peri-tumoral stroma. In addition to this extracellular role, LOX appears to take part in a range of intracellular functions, including: cell proliferation, motility and migration, chemotaxis, cell adhesion, differentiation and transcriptional gene regulation.[32–36] Hence, the tumor-suppressive functions of LOX may not be limited to the ECM.

However, paradoxical roles have been reported for LOX, functioning either as a tumor suppressor or metastasis promoter. LOX upregulation has been reported in metastatic and/or invasive BC cell lines, with invasion prevented by B-aminopropionitrile, an inhibitor of LOX.[54–57] LOX has been suggested to be a tumor-secreted factor required for ‘pre-metastatic niche’ formation.[58,59] Hypoxia and re-oxygenation appear to drive poorly invasive BC cells towards a more aggressive phenotype by LOX up-regulation.[60–63] LOX-mediated collagen cross-linking and tissue stiffening have recently been demonstrated to promote focal adhesions and tumor progression in-vivo using a mouse model of BC.[64] In keeping with this, inhibition of LOX has been demonstrated to eliminate BC metastases in a mouse model.[61,62] Furthermore, one study found LOX expression to be very low in normal breast tissue, increased in primary BC and the majority of recurrent BC, including distant metastases.[57] Interestingly, intracellular localization of LOX was noted in metastatic disease, in comparison to a stromal distribution in normal tissue. Other studies have also found LOX expression to be associated with metastasis and poor survival in BC patients.[61,62,65]

In order to reconcile these conflicting activities of LOX, it has been suggested that function may be dependent upon cellular location, cell type and transformation status.[42,44,55,61,62,66–68] The mapping of function to distinct domains of LOX and its various forms, including Pro-LOX and LOX-PP, will provide further mechanistic insights.[67–70] Indeed, LOX-PP and Pro-LOX have been demonstrated to exert opposing effects to mature LOX on tumor progression and invasion.[70] Interestingly, Rucker *et al*. have put forward a hypothesis-linking dietary copper-levels to dynamic and proportional changes in LOX activity in connective tissue.[71]

Limitations of the present study included the use of background parenchyma from BC patients to provide ‘normal tissue’ for comparison. Ideally, such material should be derived from patients without BC in order to avoid any ‘field change’, which may exist within cancer bearing tissues. Although the sample size and follow-up period were substantial, it is possible that a larger cohort, particularly with regard to late-stage patients, may have influenced several results which approached, but failed to reach, statistical significance. In addition to the measurement of mRNA transcript levels, quantitative analysis of protein expression should be undertaken to ensure concordance and determine the relationship between decreased mRNA expression and functional differences in enzyme activity. Localization experiments involving in-situ hybridization, immuno-histochemistry and confocal microscopy could be used in future studies to link expression profiles with cell type within tumor specimens and evaluate co-localization with associated molecules and markers of invasiveness and metastatic competence.

## CONCLUSION

CHIP expression is significantly associated with more favorable prognostic parameters in BC, including tumor grade, TNM stage and NPI. Furthermore, CHIP expression is a significant predictor of OS. LOX expression is significantly associated with an improved NPI. In addition to the prognostic utility of these molecules, mechanistic insights into their tumor suppressor function may offer potential for novel therapeutic strategies.

## AUTHOR’S PROFILE

**Mr. Neill Patani**, BSc(Hons) MBBS(Hons) MRCS(Eng.) Specialty Registrar in General Surgery, North-West Thames, London Deanery

**Prof. Kefah Mokbel**, MB,BS MS FRCS Consultant Breast Surgeon at St. George’s & The Princess Grace Hospitals Professor of Breast Cancer Surgery (The Brunel Institute of Cancer Genetics & Pharmacogenomics) Reader in Breast Surgery (St. George’s University of London) President of Breast Cancer Hope Charity: www.breastcancerhope.org.uk

**Prof. Robert Newbold**, Professor of Cancer Genetics and Director Brunel Institute of Cancer Genetics and Pharmacogenomics

**Prof. Wen Jiang**, MB, BCh, MD Professor of Surgery and Tumour Biology Head, Metastasis & Angiogenesis Research Group University Department of Surgery Wales College of Medicine Cardiff University
